# The Karnataka Anemia Project 2 — design and evaluation of a community-based parental intervention to improve childhood anemia cure rates: study protocol for a cluster randomized controlled trial

**DOI:** 10.1186/s13063-015-1135-x

**Published:** 2015-12-30

**Authors:** Arun S. Shet, Merrick Zwarenstein, Maya Mascarenhas, Arvind Risbud, Salla Atkins, Neil Klar, Maria Rosaria Galanti

**Affiliations:** Hematology Research Division, St. Johns Research Institute, Bangalore, India; Department of Medical Oncology, St. Johns Medical College and Hospital, Bangalore, India; Department of Family Medicine, Schulich School of Medicine and Dentistry, Western University, London, Ontario Canada; Department of Epidemiology & Biostatistics, Schulich School of Medicine and Dentistry, Western University, London, Ontario Canada; MYRADA, Bangalore, India; Department of Public Health Sciences, Karolinska Institutet, Stockholm, Sweden; Centre for Epidemiology and Community Medicine, Stockholm Health Care District, Stockholm, Sweden

**Keywords:** Iron deficiency anemia, Hemoglobin, Cluster randomization, Controlled trial, Counseling, Evaluation

## Abstract

**Background:**

Childhood anemia is highly prevalent worldwide. Improving the hemoglobin level of preschool age children could yield substantial benefits in cognitive and psychosocial development and overall health. While evidence-based recommendations for reducing childhood anemia in high anemia prevalence countries are available, there is no experimental evidence of community centered education and counseling programs, as a route to improved acceptance of iron supplements, demonstrating beneficial effects on anemia outcomes. We report on the evaluation protocol of a complex educational intervention led by the community lay health worker (LHW) and delivered to mothers of 12–59-month-old anemic children living in and visiting village day care centers in a large district of southern India.

**Methods and design:**

The study is designed as a cluster randomized controlled trial. The intervention is based on the social cognitive theory and aims to promote among mothers, anemia awareness, dietary modifications to increase iron intake in the child, and recognition of the need for enhanced adherence to supplemental iron in the anemic child. From 270 eligible villages in the study area, a sample of 60 villages will be randomized to intervention [*n* = 30] or to treatment as usual [*n* = 30] of the study. LHWs in the intervention arm will be trained to administer the following intervention components to mothers of anemic children: 1] monthly distribution of Iron and folic acid (IFA) supplements to mothers of anemic children, and 2] five monthly counseling sessions of mothers of anemic children covering: a] anemia awareness education b] IFA adherence counseling and assessment, c] dietary modification to improve iron intake, and d] hygiene and sanitation. LHWs in the control arm will distribute IFA to mothers of anemic children as in the intervention arm but will not provide monthly education and counseling support. The primary outcome is the difference between the two experimental groups in anemia cure rates of children found to be anemic at baseline. Secondary outcomes, assessed as differences between all participants in both experimental groups, are: change in mothers’ knowledge regarding anemia; 24 hour dietary iron intake; net improvement in individual hemoglobin values; serum ferritin; and the difference in overall cluster level childhood anemia prevalence. All outcomes will be measured 6 months after the start of the intervention. Multilevel linear and logistic regression models will be used to analyze differences between intervention and control groups in outcome variables.

**Discussion:**

This trial is designed to evaluate the effectiveness of an intervention intended to improve anemia cure rates in anemic children living in villages of Chamarajnagar, Karnataka a large district in south India. The extensive study of secondary endpoints will be used to identify possible weak points in the compliance to intervention delivery and uptake. This evaluation is one of the few large randomized trials evaluating the impact of an education and counseling intervention to reduce childhood anemia prevalence.

**Trial registration:**

This trial was registered with ISRCTN.com (identifier: ISRCTN68413407) on 17 September 2013.

**Electronic supplementary material:**

The online version of this article (doi:10.1186/s13063-015-1135-x) contains supplementary material, which is available to authorized users.

## Background

Over 1.6 billion people worldwide suffer from anemia and approximately 80 % of the burden of this disorder is borne by individuals living in South Asia and Africa [1–3]. Anemia is associated with a significant economic burden [4], accounts for 68.4 million years lived with disability (8.8 % of total for all conditions), increased maternal and perinatal mortality, and contributes to global mortality [5–7]. The prevalence of anemia in India is particularly high, where 50 % of reproductive age women, 59 % of pregnant women, 25 % of men, 40 % of adolescent girls, and 70 % of children under five years are anemic [8, 9]. The etiology of childhood anemia in limited resource settings is multifactorial [10–12], but in India it is mainly attributable to iron and other micronutrient deficiencies [6, 9, 13–15]. Iron deficiency anemia (IDA) is associated with cognitive and psychomotor retardation in children [16, 17], and trials of iron supplements in iron deficient children demonstrate improved outcomes [18]. The major cause of IDA in India is inadequate iron intake due to both low dietary iron content of food [19] and inadequate dietary animal protein [20]. In this context, childhood anemia appears to be mostly influenced by maternal anemia during pregnancy [8], poor nutritional and dietary iron intake [9, 19], and a combination of adverse socioeconomic factors [9].

Public health strategies addressing IDA have concentrated efforts on improving dietary iron intake by promoting population-based iron supplementation and diversification of diets, and by improving the iron content of food by fortification [21, 22]. These efforts have successfully reduced anemia prevalence in other Asian settings [20], but such benefits have not accrued in India [23, 24]. In line with WHO recommendations, the Indian government iterated the National Nutritional Anemia Control Programme (NNACP), which featured the use of iron supplements [21, 24]. More recently, launching of the national Iron + initiative by the Indian government has made anemia control more comprehensive by including an emphasis on nutrition [25]. Globally, however, challenges to childhood anemia control remain [26]. In India, inadequate procurement and distribution of iron and folic acid (IFA)[27], a perceived lack of adequate lay health worker (LHW) support in the village [27–29], and unfounded beliefs and psychological issues among individuals [9, 27] appear to hamper effective childhood anemia control. Previous research has shown that LHW-led education interventions are effective at promoting dietary modification, complementary feeding, and immunization uptake among rural communities [30–35]. We hypothesized that an educational and counseling intervention with a strong parental component specifically to reduce knowledge gaps among mothers, improve parental self-efficacy, and enhance adherence of the anemic child to (IFA would achieve better anemia cure rates than treatment as usual. This paper describes the study protocol for the Karnataka Anemia Project 2, which evaluates the effectiveness of a community LHW-led educational and counseling intervention delivered to mothers of anemic children residing in villages located in a province of southern India. We follow the CONSORT statement for reporting of cluster randomized trials [36, 37].

### Design and Methods

#### Study design and setting

The study is designed as a cluster randomized controlled trial (CRCT) set in the Chamarajnagar taluk, Chamarajnagar district of Karnataka state, India. Karnataka, a south western state with a population of 61 million (7.1 million of which are children 0–6 years of age) is the ninth most populated state in India (2011 census) [38]. The study area, Chamarajnagar district, is located in a rural setting with a predominantly agrarian economy and an average annual household income of Indian rupees (INR) 22 006 (US $478) reflecting state and national averages. The Karnataka average annual household income is INR 26 123 (US $567), and the Indian overall average annual household income is INR 25 825 (US $561). Literacy rates are slightly higher (total = 75 % M = 82 %; F = 66 %) than the national average.

In India, the Integrated Child Development Service (ICDS) runs a network of village-based child care centers (ADCs), caring for children up to age 6 years and potentially offering the setting in which to deliver health interventions to mothers of registered children [35]. The LHW in charge of the ADC is an integral component of the health system whose routine work includes the maintenance of childhood immunization records, health education for mothers, as well as kindergarten education for all village children. From the age of 36 months, children attend the ADC and receive day care services, education, and nutritional supplements under a government funded ICDS scheme. Although children younger than 36 months are registered in the ADC, for practical reasons they are not encouraged to attend. Typically the ADC LHW lives in the village, has a good relationship with mothers of children below 6 years of age, and an overall good standing in the community.

**Fig. 1:**
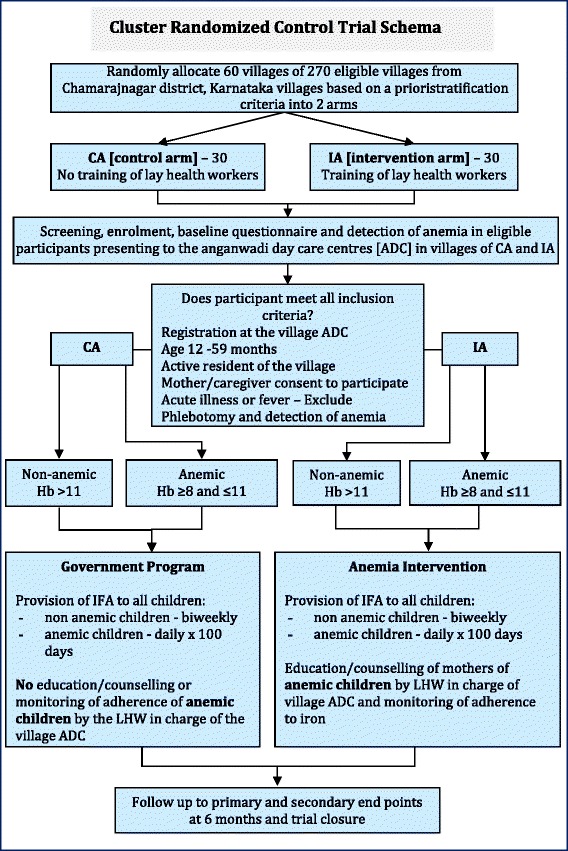
Study schematic of the Karnataka Anemia Project 2 study to evaluate a community-based parental intervention to improve childhood anemia cure rate

In this trial, a cluster (randomization unit) will be defined as a village in the Chamarajnagar subprovince that is randomly allocated to one of the study arms together with the ADC or (if the village has more than one ADC) the selected ADCs belonging to that village, and the corresponding LHW in charge of the ADC. Using a computerized random number generator [39], 60 villages from a total of 270 eligible villages will be randomly selected. The villages will be stratified based on the number of <6 year-old children listed in the ADC registers as resident in each village. After stratification, the selected villages will be assigned to intervention and control arms of the trial using a 1:1 ratio, in order to ensure equal representation of both strata to both arms. The observational units consist of children aged 12–59 months registered in the randomized villages and their mothers/caregivers, eligible for the study. We plan to recruit all eligible children (Fig. 1).

### Participants

All children aged 12–59 months, residing in the village and registered in the village ADC, will be eligible to participate in the trial.

#### Inclusion criteria

For enrollment, children should be accompanied by their mother or caregiver. Mothers/caregivers of participants in the intervention arm should consent to receive LHW-led intervention and intend to reside in the village for at least 6 months after enrollment in the trial.

#### Exclusion criteria

Children with severe anemia defined as Hb ≤ 7.9 gm/dl for the purpose of this trial [40] will be excluded and referred to the primary health center/first referral unit for assessment and medical management. Children with a reported history of an active infection or fever >101 °F will also be excluded from participation and referred to the primary health center/first referral unit for management as usual.

### Intervention

#### Rationale for the intervention and its development

**Fig. 2:**
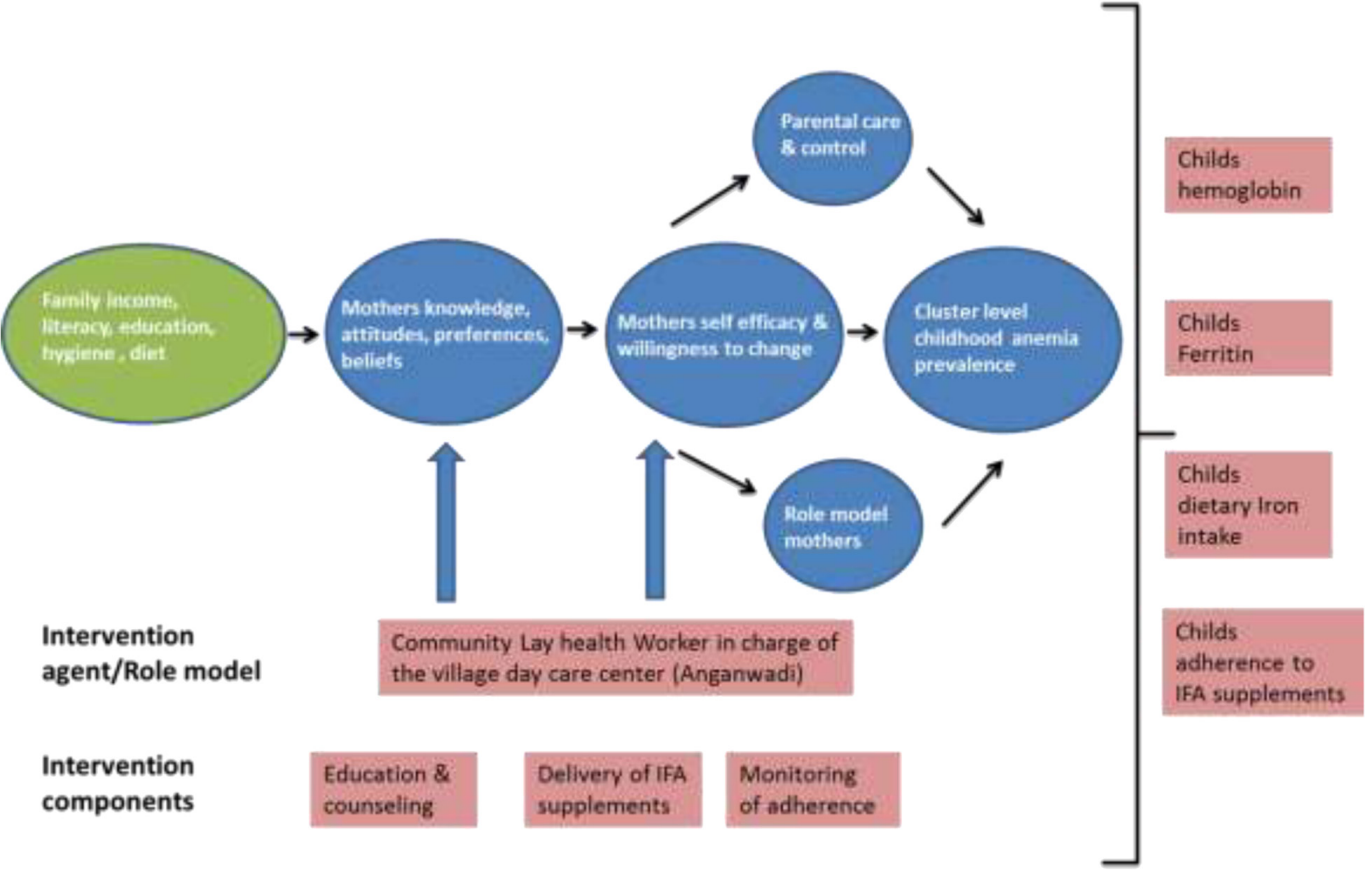
Hypothesized causal pathway of the effect of the intervention and its outcome measures

In the planning stage of the intervention, theoretical frameworks were developed according to Fraser et al [41]. In the Problem Theory, the mother’s knowledge, attitude, beliefs, care and control, role model, willingness to change, and parental self-efficacy were identified as malleable factors in order to influence the hygiene, dietary habits, and adherence to IFA supplements in their children. The design of the intervention is guided by the Social Cognitive Theory (SCT) [42], based on evidence in the published literature [23, 29, 31, 33, 34, 43, 44], and modulated based on discussions with stakeholders and policymakers in the Karnataka. According to the SCT, at least two principal sources of self-efficacy: verbal persuasion and performance accomplishment, are intended to mediate the effect of this intervention. The education of mothers about anemia, nutrition, IFA supplementation, and hygiene could foster the perception that their actions can control anemia in their children. This would lead to positive expectations about their children’s health outcomes, which, along with LHW facilitation of learning and positive reinforcement, could improve IFA adherence (Fig. 2).

Furthermore, the intervention benefits are hypothesized to spill over to the entire community. Following a socio-ecological framework [45, 46], the child’s immediate environment affected by the intervention would be the microsystem (the family) and the mesosystem (for example, parent-teacher meetings, the neighborhood, and social gatherings at the village level). At both these levels, one could expect counseled mothers of anemic children to modify the behaviors of mothers of non-anemic children, with respect to IFA consumption and dietary iron intake, thereby reducing the overall prevalence of anemia at the cluster level. Thus, the intervention is evidence based, contextually relevant, uses locally relevant resources, and has the potential to influence national policy.

#### Lay health worker training

LHWs in the intervention villages [*n* = 30] will receive training under four separate hands-on workshops over a period of 6 months. Training will occur with the use of role play, facilitation, and flip charts to help develop counseling and education skills of the LHWs. Training will take place at a location different from the LHWs’ habitual training/supervision sites, to minimize the risk of contamination. LHWs will be trained to deliver five monthly education and counseling sessions to mothers/caregivers of anemic children. Specifically, these sessions will aim to improve mothers’ knowledge regarding anemia and its consequences, the treatment of iron deficiency anemia with IFA, the importance of adherence to IFA treatments, sources of iron-rich food, diet modification behaviors, and hygiene. The possibility of contamination during LHW-supervisor monthly meetings, due to interaction between control group and intervention group LHWs, is minimal. LHWs in the control group neither receive training nor have access to training and intervention materials and will therefore be unable to replicate the intervention in their villages.

### Components of the Intervention

i) *Enrollment and screening for anemia*
  All eligible participants providing informed consent will be enrolled in the trial, provide information for a baseline questionnaire, have anthropometric measurements, and venous blood obtained by phlebotomy to screen for anemia by the research team.
ii) *Iron supplementation*
  A core preventive measure common to both trial arms is the delivery of iron supplements. All enrolled non-anemic participants are eligible for IFA supplements (tablets containing 20 mg elemental iron, 8 tablets/month) in line with National Iron + guidelines [25]. The guidelines recommend 20 mg IFA dispensed as syrup biweekly throughout the period 6–60 months and deworming for children 12 months and older. Since IFA syrup was not available in Karnataka, for the purpose of this trial, IFA is provided as 20 mg tablets. All enrolled participants detected with anemia (Hb ≥ 8 and ≤ 11 gm/dL) [40] in both arms of the trial are eligible for the therapeutic IFA dosage to control iron deficiency anemia (tablets containing 20 mg elemental iron, 20 tablets/month for 5 months). All subjects with acute infections or fever will be excluded during enrollment to the trial. Furthermore, during dispensation of IFA tablets, LHWs will advise mothers to withhold these during periods of fever or acute infections.
iii) *Education and counseling*
  Only mothers/caregivers of anemic children (Hb ≥ 8 and ≤ 11 gm/dL) in the intervention arm will receive five monthly education and counseling sessions delivered by the LHW over the 6-month intervention period (Fig. 1).
iv) *Monitoring of adherence to IFA*
  From the second counseling visit onward, LHWs in the intervention arm perform monthly pill counts to assess adherence to IFA dispensed only to mothers/caregivers of anemic children in the previous month and document the side effects of IFA, if any. In accordance with precautions in the guidelines, LHWs will advise mothers to withhold supplemental and treatment IFA during periods of fever or acute infections. A similar monthly assessment of adherence for mothers of anemic children in the control arm will not be performed. However, at the end of the 6 months of intervention, an assessment adherence to IFA in the previous 30 days will be performed for all participants in both arms of the trial.

### Outcomes

The primary outcome will be the difference in anemia cure rate between the experimental arms at the end of 6 months from the intervention’s start, in children found to be anemic at baseline. In addition, we will assess several secondary outcomes occurring along the hypothesized causal pathway of the intervention effect (Fig. 2), including: 1) difference in changes of knowledge and practice of mothers of anemic children from baseline to follow-up, 2) differences in the estimated 24-hour dietary iron intake among anemic participants exposed and not exposed to the intervention, 3) difference in net improvements in individual hemoglobin values between baseline and 6 month follow-up among anemic children exposed and not exposed to the intervention, 4) net improvements in mean ferritin values among anemic participants exposed and not exposed to the intervention, and, 5) difference in the cluster level anemia prevalence between experimental arms. Additional process indicator data include evaluation of the proportion of expected IFA doses delivered to children in the study (adherence to iron treatment), maintenance of LHW records of delivery of the intervention and IFA, and recording of the incidence of side effects of IFA supplements in children. Qualitative research on the acceptability, perceptions, and experiences of the LHWs regarding the training for and the actual delivery of the intervention will be conducted using focus group discussions. These data will help identify key assumptions and limitations of underlying intervention feasibility and sustainability and scaling-up, particularly when involving LHWs at the national level.

### Protocol of recruitment

From the list of all children < 6 years registered in each village by the LHW in charge of the ADC, the research team will generate an eligible 12–59 month child list. Using this list, the LHW will mobilize participants and their mothers to the village ADC for study enrollment. Recruitment will occur at the village ADC and last for approximately 2 days. Additional house visits will be made on recruitment days to identify reasons for non-attendance of participants on the list (for example, those who are too sick, traveling, or migrating] and to help mobilize those who forgot to visit the ADC. Mothers/caregivers of all eligible children, who after being provided information about the trial indicate willingness to participate and an intention to live in the village for at least 6 months after enrollment, and those in the intervention arm indicating a willingness to receive LHW-led counseling will be enrolled after providing written informed consent (Fig. 1). Where recruitment is <80 % of the eligible list, an additional recruitment day will be allocated. Children who do not fulfill the inclusion criteria during recruitment sessions are not eligible for future recruitment.

### Protocol of data collection

At study entry, a baseline questionnaire will record participant information. An additional file shows this in more detail [see Additional file 1]. Participants will also have a baseline sample of anticoagulated venous blood obtained to detect anemia and measure serum ferritin. All recruited participants will have a hemoglobin value estimated using an automated cell counter (Sysmex 405, Transasia laboratory systems, Japan). Within a few days the research team will provide mothers/caregivers with results of the hemoglobin values and anthropometry, and cluster LHWs with a list of anemic and non-anemic children. The list will permit LHWs in each cluster to organize the delivery of dose appropriate IFA to anemic and non-anemic children and to identify mothers of anemic children for delivery of monthly education and counseling.

The date of delivery of the first month’s supply of IFA to the mothers of children in a cluster will herald the start of the intervention in that cluster. LHWs will deliver IFA to mothers of non-anemic children at the ADC during routine monthly visits. LHWs will typically deliver IFA together with education and counseling to mothers of anemic children at their homes. From the second visit onward, adherence data will be collected by the LHW, and the remaining IFA pills/empty strip will be obtained from the mother. In the control arm, LHWs will deliver IFA as usual in the setting of the ADC and will not deliver education and counseling to mothers of anemic children or collect monthly IFA adherence data. Approximately 6 months from the day of delivery of the first IFA supplement, the research team will perform a follow-up visit to collect 24-hour dietary recall data, anthropometry, and information on adherence to IFA in the previous month from all enrolled participants. During this time, a repeat phlebotomy to estimate hemoglobin is performed for all participants in the cluster, and the trial is completed.

Questionnaires prior to and after the intervention from mothers of anemic children provide information about the impact of LHW-delivered education and counseling on the mother’s knowledge regarding anemia and its effects on the child. The mother’s knowledge and practice questionnaire was explored by pilot testing it in the field for ease of administration and understanding. Assessment of 24-hour dietary iron intake at baseline and at the end of the intervention will provide information to estimate the effect of this intervention on promoting dietary diversification and improving dietary iron content. Finally, assessments of ferritin, a serum marker of iron stores, will provide information on whether actual improvements have been made in the total body iron stores of anemic children.

### Statistical methods

#### Statistical power and sample size calculation

Primary analyses are limited to children with anemia at enrollment. At the end of 6 months, a difference of at least 12 % in the cure rate of anemic children is required if the intervention is considered to have sufficient public health utility to warrant its cost and effort [47–49]. We estimate that the 12 % reduction in proportion of anemic children at 6 months would occur due to a cure rate of 30 % in the control group (IFA alone) and 42 % in the experimental arm where children receive IFA plus the community-based parental intervention. To detect a 12 % difference in 6-month anemia cure rates at an alpha level of 0.05 for a two-tailed test, with 80 % power, a total unadjusted sample size of 500 children with anemia is required, which after adjustment for clustering (design effect = 2.2) results in a sample size of 1,100 children with anemia. The design effect assumes an intracluster correlation coefficient (ICC) of 0.05 and 25 participating children with anemia per village. The anticipated degree of intracluster correlation was selected based on results from several recent trials of interventions on anemia [47–49]. We further allow for a 10 % loss to follow-up of children identified with anemia at baseline, yielding a final sample size of 610 children with anemia per arm (1,220 in all). We will recruit 30 clusters per trial arm to accommodate potential losses of clusters if LHWs should stop working, resulting in loss of an entire cluster. Thus, power will be greater than 80 %. Analyses will also consider prevalence of anemia at 6 months among all participating children regardless of their baseline anemia status. Based on an estimated prevalence of 50 % anemia at baseline in each village, the number of children required will be 1,220 in each trial arm.

#### Statistical analysis

The primary analysis will follow the intention-to-treat principle, whereby all children regardless of whether they received the full intervention or not will be included in the analysis of the group to which they were randomized. Data will be analyzed at the individual level adjusting for clustering using generalized estimating equation (GEE) extensions of logistic regression for presence or absence of anemia while similar extensions of linear regression will be used to model individual hemoglobin levels [50]. All models will adjust for the stratification factor (the number of children per village). The effects of explanatory variables such as age, sex, socioeconomic status, literacy, nutritional parameters, and maternal anemia status will also be explored. Secondary analyses will consider the effect of adjustment for these potential explanatory variables on the estimated intervention effect using multivariable regression models. All tests will be declared statistically significant at the 0.05 level (two-tailed).

### Ethical issues

#### Ethical approval

The study was approved by the St. Johns National Academy of Health Sciences Institutional Ethical Committee (IEC 115/2012).

#### Information and informed consent

Information about the trial is provided to community leaders and mothers through the LHW. Written and verbal information about the trial is provided in English and Kannada (the local language). Documents translated into Kannada are validated through back translation. All participant mothers/care providers are provided with study information prior to enrollment for review. Individual informed consent is obtained from mothers/care providers of selected children, and is recorded by signature or thumbprint. Mothers/care givers will also be informed that participation of their child in the study is completely voluntary and that they may withdraw from the study at any time.

#### Monitoring of side effects and trial supervision

Details regarding side effects to IFA supplements will be collected by the LHW and notified to the research team. In the event of side effects that are deemed related to IFA (either by the LHW or the research team), participants are advised by the LHW to discontinue IFA and report to the primary health center for evaluation. Since the intervention under comparison is non-invasive, the IFA supplements in the protocol follow national recommendations, and the setting is not a malaria endemic region, the occurrence of serious adverse events (SAEs) is unlikely. An interim analysis of the trial will take place when all subjects have been recruited and all 30 clusters have completed 3 months of delivery of the intervention. This analysis will be conducted in a blinded manner by an independent data safety and monitoring board (DSMB) consisting of experts in clinical medicine, epidemiology, and statistics. The primary aim of the interim analysis is to assess if the intervention arm shows a clearly better, or worse, outcome than the control arm as well as to evaluate SAEs.

## Discussion

This is the first cluster randomized control trial (CRCT) designed to evaluate an intervention that includes both lay health workers and parents to complement India’s existing national health policy for combating anemia in children, one of the most pernicious and common nutritional health burdens among individuals in resource-limited settings [3, 6, 10, 51]. The present evaluation adopts a theory-based approach to investigate the causal chain through which the proposed intervention is expected to have its impact (Fig. 2). We employ qualitative methods to understand the perceptions and experiences of LHWs actually delivering the intervention and the context and process of the interventions delivery [52]. Results from this contextually relevant intervention will be used to establish whether it is possible to improve anemia cure rates in anemic children in India with relatively limited efforts targeted to the mothers of such children.

The CRCT design, whereby groups of individuals are randomly allocated to different interventions, has the potential to provide unbiased estimates of the impact of interventions delivered at the community level (in this case children in a village eligible to attend the village day care). A major strength of this trial is that it attempts to maximize participation at both the cluster and individual levels. For instance, LHWs in both arms of the study will be provided with training, support, and resources to achieve intervention goals, and will encourage cluster-level participation to minimize drop-out rates. Furthermore, randomization will be an inclusive process with active involvement and participation of the LHWs. During recruitment, the research team will actively support LHWs during periods of stress (wage-related agitations, unscheduled ADC closures for local holidays, and breaks during which LHWs perform other tasks such as immunization). The research team will perform door-to-door visits for eligible children who failed to attend the ADC after mobilization by the LHW to identify reasons for the mothers’ lack of attendance. Analysis of all this data will provide clarity regarding participation in this trial and could address concerns related to selection bias.

Anemia has been a public health problem for several decades [3, 6] and is in great need of effective control programs. India bears a large portion of the burden of anemia in Asia, with close to 100 million anemic children [2, 6]. The need for effective intervention studies addressing anemia is particularly relevant to rural Indian communities, where the prevalence of anemia is highest. However, there is an increasing appreciation that such studies should not only evaluate if interventions work, but also how they work, thereby enhancing the policy implications of such evaluations [52, 53]. In order to maximize policy relevance, it is essential to understand the context of the research, the related policymaking processes and to engage key stakeholders. To achieve this, we intend to continuously engage with the health authorities at state, provincial, and district levels. Inadequate IFA supplies have been previously noted to be a major limitation to the success of anemia control in low-middle income settings [28]. Thus, instead of procuring IFA commercially, the Karnataka state health officials will be involved and the state infra-structure will be utilized to ensure that IFA is made available for the trial. These efforts could eventually lead to greater sensitization of the health department regarding IFA availability and recognition of the need for enhanced LHW capacity to screen and manage anemia in Karnataka. Conducting research in rural communities, especially in an Indian context, raises a number of practical and logistic issues. The research team has partnered with an experienced field research agency that has an established track record of development research in the Chamarajnagar district. This team will work collaboratively, coordinate with the government, involve community leaders from villages in the study area, and make participants stakeholders in their healthcare, all of which will contribute to the successful conduct of the trial.

Indeed, the novelty of this cluster RCT consists of its real-life setting evaluation of education and counseling combined with monitoring of parental compliance in connection with the delivery of IFA supplements to attenuate the public health problem of childhood anemia. All intervention components will be delivered by a community LHW, therefore relying on local human resources. Furthermore, the intervention will use healthy local dietary practices to positively influence the nutritional behaviors of children and parents. If successful, such an approach could profoundly shift the attention of anemia control policy towards a diet-focused rather than a supplementation-focused approach. Once effectiveness in real-life conditions is ascertained, all the above features of the intervention will collectively ensure high potential for sustainability and scaling-up of the intervention. The intervention may lead to improved hemoglobin values and reduced anemia prevalence among 12–59-month children in the short term [18, 26] and could result in better cognitive outcomes in these children over the long term [18, 54–56]. If effective, the intervention could be implemented on a larger scale to determine if such local effects may have a wider positive health effect nationally. Final results from this intervention trial are expected in mid-2016.

## Trial status

The trial commenced recruitment in November 2014 and intends to complete recruitment in July 2015.

## Additional file

Additional file 1:
**Baseline questionnaire.** (PDF 287 kb)

## Abbreviations

ADC: Anganwadi day care center
CHW: community health worker
Hb: hemoglobin
IDA: iron deficiency anemia
IFA: iron and folic acid
Iron + Initiative: National Iron plus initiative
LHW: lay health worker
NNACP: National Nutritional Anemia Control Programme
