# Supplementary material for: The Karnataka Anemia Project 2 — design and evaluation of a community-based parental intervention to improve childhood anemia cure rates: study protocol for a cluster randomized controlled trial
Source: Trials. 2015 Dec 30;16:599. doi: 10.1186/s13063-015-1135-x (PMC4697328; doi:10.1186/s13063-015-1135-x)
Supplement: Additional file 1: — Baseline questionnaire. (PDF 287 kb) [file 13063_2015_1135_MOESM1_ESM.pdf]

BASELINE ASSESSMENT FORM

(Completed by research team at baseline for all participants)

☐☐☐	☐☐☐☐	Date: ☐☐ ☐☐ ☐☐☐☐
Village ID<br>ಹಳ್ಳಿಯ ಸಂಖ್ಯೆ	PID<br>ಸಂಖ್ಯೆ	(dd) (mm) (yyyy)<br>ದಿನಾಂಕ:

Village name: ______________________	Activity done	Interview/Labs		QC	Data Entry 1	Data Entry 2
	Person's Initials					
	Date					

A. Participant details: ಭಾಗವಹಿಸಿದವರ ಮಾಹಿತಿ:

A1	Child's name:<br>ಮಗುವಿನ ಹೆಸರು:	A2	Sex: ಲಿಂಗ:<br>☐ 1.Male ☐ 2.Female
A3	Date of birth: ಹುಟ್ಟಿದ ದಿನಾಂಕ<br>____ / ____ / ________<br>dd mm yyyy	A4	Age: ವಯಸ್ಸು:<br>______yrs ______months<br>(eg:"2yr 3mon")
A5	Name of care-giver: ಪೋಷಕರ ಹೆಸರು	A6	Relationship of care-giver to child:<br>ಪೋಷಕರ ಜೊತೆಗಿನ ಸಂಬಂಧ:<br>☐ 1. Mother (ತಾಯಿ)<br>☐ 2. Others, specify: ______________<br>ಇತರೆ: ನಮೂದಿಸಿ:

B. Socio-demographic questions (mother): ಸಾಮಾನ್ಯ ಮಾಹಿತಿ (ಜನಸಂಖ್ಯೆಗೆ ಸಂಬಂಧಿಸಿದಂತೆ)

B1	Mother's age :<br>ತಾಯಿಯ ವಯಸ್ಸು:<br>___________(e.g. "25 yr")<br>☐ Deceased (ಮರಣವೊಂದ್ದಿದರೆ)	B2	Mother's religion: ತಾಯಿಯ ಧರ್ಮ:<br>☐ 1.Hindu ☐ 2.Muslim ☐ 3.Christian<br>1. ಹಿಂದೂ 2. ಮುಸ್ಲಿಂ 3. ಕ್ರಿಶ್ಚಿಯನ್<br>4. ಇತರೆ: ನಮೂದಿಸಿ:<br>☐ 4. Other, specify: _________________
B3	No. of years of schooling:<br>___________ years<br>ನೀವು ಎಷ್ಟು ವರ್ಷ ಶಾಲೆಗೆ ಹೋಗಿದ್ದೀರಾ?	B4	Can you read: ☐ 1. Yes ☐ 2.No<br>ನಿಮಗೆ ಓದಲು ಬರುತ್ತದೆಯೆ: ೧. ಹೌದು ೨. ಇಲ್ಲ<br>Can you write: ☐ 1.Yes ☐ 2.No<br>ನಿಮಗೆ ಬರೆಯಲು ಬರುತ್ತದೆಯೆ: 1. ಹೌದು 2. ಇಲ್ಲ

C. Anemia risk factors (mother):

C1	How many pregnancies have you had so far?<br>ನೀವು ಇಲ್ಲಿಯ ವರೆಗೆ ಎಷ್ಟು ಭಾರಿ ಗರ್ಭಿಣಿಯಾಗಿದ್ದೀರ?<br>Number of pregnancies: __________<br>No. of abortions/still birth: __________ (if any)	C2	Could you tell me the birth order of this child among live births?<br>ಜೀವಂತವಾಗಿ ಹುಟ್ಟಿದ ಮಕ್ಕಳಲ್ಲಿ ಈ ಮಗು ಎಷ್ಟನೆಯದು?<br>Number of live births: ___________<br>Birth order: _________

D. Anemia risk factors (child) ರಕ್ತಹೀನತೆಯ ಲಕ್ಷಣಗಳು (ಮಗು)

D1	Did you breast feed this child?<br>ನಿಮ್ಮ ಈ ಮಗುವಿಗೆ ಎದೆ ಹಾಲು ಕುಡಿಸಿದ್ದೀರಾ?<br>☐ 1. Yes ☐ 2. No *(If 'No' goto D7)*<br>1. ಹೌದು 2. ಇಲ್ಲ *(ಇಲ್ಲ ಎಂದರೆ D7 ಕ್ಕೆ ಹೋಗಿ)*		
D2	For how many months did you give the child breast milk alone, with no other foods (exclusive breastfeeding)?<br>____________ months<br>ನಿಮ್ಮ ಮಗುವಿಗೆ ಬೇರ ಯಾವುದೆ ರೀತಿಯ ಆಹಾರವನ್ನು ನೀಡದೆ ಬರಿ ಎದೆ ಹಾಲನ್ನು ಎಷ್ಟು ತಿಂಗಳವರೆಗೆ ನೀಡಿದ್ದೀರಾ?	D3	Are you still breastfeeding this child?<br>ಈಗಲು ನೀವು ಈ ಮಗುವಿಗೆ ಎದೆ ಹಾಲು ಕುಡಿಸುತ್ತಿದ್ದೀರಾ?<br>☐ 1. Yes ☐ 2. No<br>*(If 'No', goto D6)*<br>1. ಹೌದು *(ಇಲ್ಲ ಎಂದರೆ D6 ಕ್ಕೆ ಹೋಗಿ)*<br>2. ಇಲ್ಲ
D4	How many times do you breast feed the child in a day?<br>ಒಂದು ದಿನದಲ್ಲಿ ಸುಮಾರು ಎಷ್ಟು ಬಾರಿ ಈ ಮಗುವಿಗೆ ಎದೆಹಾಲು ಕುಡಿಸುತ್ತಿರೀ?<br>☐ 1. 2 times<br>☐ 2. 2-4 times<br>☐ 3. >4 times	D5	How long do you feed the baby on an average in a day?<br>ಒಂದು ದಿನದಲ್ಲಿ ಸಮಾನ್ಯವಾಗಿ ಎಷ್ಟು ಸಮಯ ಈ ಮಗುವಿಗೆ ಎದೆಹಾಲು ಕುಡಿಸುತ್ತಿರೀ?<br>☐ 1. 5-10 min<br>☐ 2. 10-20 min<br>☐ 3. >20 min

PID ☐☐☐☐

D6 For this child at what age did you stop breastfeeding altogether?

Age : ____________ months

ನಿಮ್ಮ ಈ ಮಗುವಿಗೆ ಯಾವ ವಯಸ್ಸಿಗೆ ಎದೆ ಹಾಲು ಕುಡಿಸುವುದನ್ನು ನಿಲ್ಲಿಸಿದ್ದೀರಾ?

ವಯಸ್ಸು___________ತಿಂಗಳುಗಳಲ್ಲಿ.

D7 At what age did you first give other foods for this child?

Age : ____________ months

ನಿಮ್ಮ ಈ ಮಗುವಿಗೆ ಯಾವ ವಯಸ್ಸಿನಲ್ಲಿ ಪೂರಕ ಆಹಾರವನ್ನು ಕೊಡಲು ಪ್ರಾರಂಭಿಸಿದ್ದೀರಿ?

ವಯಸ್ಸು___________ತಿಂಗಳುಗಳಲ್ಲಿ.

☐ Not yet started (ಇನ್ನೂ ಪ್ರಾರಂಭಿಸಿಲ್ಲ)

D8 Has the child ever tested for anemia(weakness) before?

☐1.Yes ☐ 2. No ☐3. Unsure

ನಿಮ್ಮ ಮಗುವಿಗೆ ಇಲ್ಲಿಯವರೆಗೆ ಯಾವುದಾದರೀತಿಯ ರಕ್ತಹೀನತೆಯ (ಸುಸ್ತು, ಆಯಾಸ) ಪರೀಕ್ಷೆಯನ್ನು ಮಾಡಿಸಿದ್ದೀರಾ?

1. ಹೌದು 2. ಇಲ್ಲ 3. ನೆನಪಿಲ್ಲ

D9 Has the child ever received iron/ folic acid tablets or syrup?
(*Interviewer prompt* : visual cue)

☐1. Yes ☐ 2. No ☐ 3. Unsure

(If 'Unsure/No', goto D13)

ನಿಮ್ಮ ಮಗುವಿಗೆ ಕಬ್ಬಿಣಾಂಶದ ಮಾತ್ರೆ/ಸಿರಪ್‌ನ್ನು ನೀಡಿದ್ದೀರಾ? 1. ಹೌದು 2. ಇಲ್ಲ 3. ನೆನಪಿಲ್ಲ

(ಇಲ್ಲ/ ಗೊತ್ತಿಲ್ಲ ಎಂದರೆ D13ಕ್ಕೆ ಹೋಗಿ)

D10 If yes, from whom? ಹೌದಾದರೆ ಯಾರಿಂದ ಮಾತ್ರೆಗಳನ್ನು ತೆಗೆದುಕೊಂಡಿದ್ದೀರಿ?

☐ 1. Auxiliary nurse midwife (ಆರೋಗ್ಯ ಕಾರ್ಯಕರ್ತೆ)
☐ 2. Anganwadi worker (ಅಂಗನವಾಡಿ ಕಾರ್ಯಕರ್ತೆ)
☐ 3. Health worker at sub centre (ಪ್ರಾಥಮಿಕ ಆರೋಗ್ಯ ಕೇಂದ್ರದ ಆರೋಗ್ಯ ಕಾರ್ಯಕರ್ತೆ)
☐ 4. PHC (ಪ್ರಾಥಮಿಕ ಆರೋಗ್ಯ ಕೇಂದ್ರ)
☐ 5. From private shop, non-health worker initiated (ಖಾಸಗಿ ಔಷಧಾಲಯ, ಬೇರೆಯವರ ಬಳಿ ತೆಗೆದುಕೋಡಿರುವುದು)
☐ 6. Private doctor (ಖಾಸಗಿ ವೈದ್ಯರು)
☐ 7. Can't remember / Don't know (ನೆನಪಿಲ್ಲ / ಗೊತ್ತಿಲ್ಲ)

D11 How many tablets were given in last one year?
ಮಾತ್ರೆಗಳನ್ನು ನೀಡಿದ್ದರೆ ಕಳೆದ ಒಂದು ವರ್ಷದಲ್ಲಿ ಎಷ್ಟು ಮಾತ್ರೆಗಳನ್ನು ನೀಡಿದ್ದೀರಾ?

☐ 1. < 30 (<3 strips)
☐ 2. 30-60 (3-6 strips)
☐ 3. 60-90 (6-9 strips)
☐ 4. > 90 (>=9 strips)
☐ 5. No. of bottles:_________
☐ 6. None (ಯಾವೂದು ಇಲ್ಲ)
☐ 7. Don't know (ಗೊತ್ತಿಲ್ಲ)

D12 How many tablets did your child actually take?
ನೀವು ಮಾತ್ರೆಗನ್ನು ನೀಡಿದ್ದರೆ ನಿಮ್ಮ ಮಗು ಎಷ್ಟು ಮಾತ್ರೆಗಳನ್ನು ನುಂಗಿದೆ.

☐ 1. < 30 (<3 strips)
☐ 2. 30-60 (3-6 strips)
☐ 3. 60-90 (6-9 strips)
☐ 4. > 90 (>=9 strips)
☐ 5. No. of bottles:_________
☐ 6. None (ಯಾವೂದು ಇಲ್ಲ)
☐ 7. Don't know (ಗೊತ್ತಿಲ್ಲ)

D13	Has your child ever received Vitamin A liquid or capsules?<br>(*Interviewer prompt* : visual cue)<br>☐ 1. Yes ☐ 2. No ☐ 3. Unsure<br>*(If 'Unsure/No', goto D15)*<br>ನಿಮ್ಮ ಮಗುವಿಗೆ ಯಾವಾಗಲಾದರೂ ವಿಟಮಿನ್ – ಎ (ಇರುಳುಗುಡುತನ)ಮಾತ್ರೆ/ಸಿರಪ್ ಅನ್ನು ನೀಡಿದ್ದೀರಾ?<br>1. ಹೌದು 2. ಇಲ್ಲ 3. ನೆನಪಿಲ್ಲ<br>*(ಇಲ್ಲ, ಗೊತ್ತಿಲ್ಲ ಎಂದರೆ D15 ಹೋಗಿ)*	D14	If yes, how many times in the last year?<br>ಹೌದಾದರೆ ಕಳೆದ ವರ್ಷದಲ್ಲಿ ಎಷ್ಟು ಬಾರಿ ನೀಡಿದ್ದೀರಿ?<br>__________<br>☐ Don't know (ಗೊತ್ತಿಲ್ಲ)
D15	Has your child ever received Alben-dazole tablets? (*Interviewer prompt* : visual cue)<br>☐ 1. Yes ☐ 2. No ☐ 3. Unsure<br>*(If 'No/Unsure', goto D17)*<br>ನಿಮ್ಮ ಮಗುವಿಗೆ ಯಾವಾಗಲಾದರೂ ಜಂತುಹುಳು ನಿವಾರಕ ಮಾತ್ರೆಗಳನ್ನು ನೀಡಿದ್ದೀರಾ?<br>1. ಹೌದು 2. ಇಲ್ಲ 3. ನೆನಪಿಲ್ಲ<br>*(ಇಲ್ಲ/ಗೊತ್ತಿಲ್ಲ ಎಂದರೆ D17ಗೆ ಹೋಗಿ)*	D16	If yes, how many times in the last year?<br>ಹೌದಾದರೆ ಕಳೆದ ವರ್ಷದಲ್ಲಿ ಎಷ್ಟು ಬಾರಿ ನೀಡಿದ್ದೀರಿ?<br>__________<br>☐ Don't know (ಗೊತ್ತಿಲ್ಲ)

Immunization History (ಚುಚ್ಚುಮದ್ದಿನ ಮಾಹಿತಿ)<br>1. Card 2. PHC/Anganwadi register				
D17	Which vaccinations has your child received (tick those which have been received):<br>ಯಾವ ಯಾವ ಚುಚ್ಚುಮದ್ದುಗಳನ್ನು ನಿಮ್ಮ ಮಗುವಿಗೆ ಕೊಡಿಸಿದ್ದೀರಾ? (ಕೊಟ್ಟಿರುವ ಚುಚ್ಚುಮದ್ದುಗಳ ಬಾಕ್ಸನಲ್ಲಿ ಟಿಕ್ ಮಾಡಿ).			
	0 (birth)	BCG ☐	Hepatitis B – 0 ☐	OPV – 0 ☐
	6 weeks(1.5 mo)	DPT – 1 ☐	Hepatitis B – 1 ☐	OPV – 1 ☐
	10 weeks (2.5 mo)	DPT – 2 ☐	Hepatitis B – 2 ☐	OPV – 2 ☐
	14 weeks (3.5 mo)	DPT – 3 ☐	Hepatitis B – 3 ☐	OPV – 3 ☐
	9 months	Measles ☐	Vit A 1st Dose ☐	
	18 months	DPT Booster 1☐	OPV Booster 1 ☐	
	Vit A 2nd- 9th Dose (upto 5 years)	☐ 1 1/2y ☐ 2y ☐ 2 1/2y ☐ 3y ☐ 3 1/2y ☐ 4y ☐ 4 1/2y ☐ 5y		
	4-5 years	DPT Booster 2☐	OPV Booster 2 ☐	Others, Specify:
	Other vaccinations:	Hib ☐	Specify age/s at vaccination:	
	Other vaccinations:	Jap E ☐	Specify age/s at vaccination:	
	Other vaccinations:	__________	Specify age/s at vaccination:	

PID | | | | |

E. 24 hour dietary recall (child): ೨೪ ಗಂಟೆಗಳಲ್ಲಿ ಸೇವಿಸಿದ ಆಹಾರದ ಮಾಹಿತಿ

E1. Was yesterday a typical day in terms of what your child had to eat? ☐ 1.Yes ☐ 2. No
If 'Yes': recall yesterday's.
ನೆನ್ನೆ ನಿಮ್ಮ ಮಗು ಸಾಮನ್ಯತರಹದ ಆಹಾರವನ್ನು ತಿಂದ್ದಿತ್ತಾ?
1. ಹೌದು 2. ಇಲ್ಲ
ಹೌದಾದರೆ: ಹಿಂದಿನ ದಿನ ನೆನಪಿಸಿಕೊಳ್ಳೀ

If 'No': recall most recent typical day (write how many days ago: ____________)

ಇಲ್ಲಾವಾದಲ್ಲಿ ಸಾಮನ್ಯತರಹದ ಆಹಾರವನ್ನು ಎಷ್ಟು ದಿನಗಳ ಹಿಂದೆ ತಿಂದ್ದಿತ್ತು?

E2. Date of recall: ____/____/_______ (dd/mm/yyyy)
ನೆನೆಪು ಮಾಡಿಕೊಂಡ ದಿನಾಂಕ:

Time period of recall: From ______AM to ______AM Oil used: ___________
(ಸಮಯ) (ಎಲ್ಲಿಂದ) (ಎಲ್ಲಿವರೆಗೆ) (ಉಪಯೋಗಿಸಿದ ಎಣ್ಣೆ)

Are you vegetarian/non-vegetarian? ☐ 1.Veg ☐ 2. Non-veg ☐ 3. Veg + Egg

ನೀವು ಸಸ್ಯಹಾರಿಗಳೆ ಅಥವಾ ಮಾಂಸಹಾರಿಗಳೆ? 1. ಸಸ್ಯಹಾರಿ 2. ಮಾಂಸಹಾರಿ 3. ಸಸ್ಯ ಮತ್ತು ಮಾಂಸಹಾರಿ.

Period of consumption<br>ಆಹಾರ ತಿಂದ ಸಮಯ	Food consumed<br>ಸೇವಿಸಿದ ಆಹಾರ	Amount (gms/ml)<br>ಪ್ರಮಾಣ (ಗ್ರಾಂ/ಮಿಲಿ)
Early morning (before breakfast)/<br>ಮುಂಜಾನೆ/ತಿಂಡಿಗೆ ಮುಂಚೆ		
Breakfast/ತಿಂಡಿ		
Mid-morning / ತಿಂಡಿಯ ನಂತರ		

Period of consumption<br>ಆಹಾರ ತಿಂದ ಸಮಯ	Food consumed<br>ಸೇವಿಸಿದ ಆಹಾರ	Amount (gms/ml)<br>ಪ್ರಮಾಣ (ಗ್ರಾಂ/ಮಿಲಿ)
Lunch/ ಊಟ		
Evening/ಸಾಯಂಕಾಲ		
Dinner/ ಊಟ		
Bed time food / ರಾತ್ರಿಯ ಸಮಯದಲ್ಲಿ ಏನಾದರು ಆಹಾರವನ್ನು ತಿಂದ್ದಿತ್ತಾ?		
Any other food items during the day/ ದಿನದಲ್ಲಿ ಬೇರೆ ಏನಾದರು ಆಹಾರವನ್ನು ತಿಂದ್ದಿತ್ತಾ?		

F. Standard of Living Index Questionnaire/ *ಜೀವನಮಟ್ಟ ಸೂಚ್ಯಂಕಕ್ಕೆ ಸಂಬಂಧಿಸಿದ ಪ್ರಶ್ನೆಗಳು:*

G. Hygiene and Sanitation / ಸ್ವಚ್ಛತೆ :

	Question	Choose the appropriate answer
F1	Type of house<br>*ಯಾವ ರೀತಿಯ ಮನೆ*	☐ 1. Pucca / ಪಕ್ಕಮನೆ, ಮೊಲ್ಡ್ ಮನೆ<br>☐ 2. Semi-pucca / ಹೆಂಚಿನ ಮನೆ<br>☐ 3. Katcha / ಗುಡಿಸಲು
F2	Does this household own this house or any other house?<br>*ಎಲ್ಲದರೂ ಸ್ವಂತ ಮನೆ ಇದೆಯೇ?*	☐ 1. Yes / ಹೌದು<br>☐ 2. No / ಇಲ್ಲ

(1) **Pucca:** One which is built with a foundation, using stone or bricks with and cement, having concrete or a stone laid roof

(2) **Semi-pucca:** One house in which some cement or mortar plastering or flooring or roofing is used.

(3) **Kutcha:** A construction with more than one room and using mud walls and a thatched roof.

PID | | | | |

	Question	Choose the appropriate answer
F3	Do you have a separate room for kitchen?/ ನಿಮ್ಮ ಮನೆಯಲ್ಲಿ ಅಡುಗೆ ಕೋಣೆ ಬೇರೆಯಾಗಿ ಇದೆಯೇ?	☐ 1. Yes/ ಹೌದು<br>☐ 2. No/ ಇಲ್ಲ
F4	What type of fuel does your household mainly use for cooking?<br>ನಿಮ್ಮ ಮನೆಯಲ್ಲಿಅಡುಗೆ ಮಾಡಲು ಯಾವ ಇಂಧನವನ್ನು ಬಳಸುತ್ತೀರಾ?	☐ 1. Wood/ಕಟ್ಟಿಗೆ<br>☐ 2. Crop residues/ಬೆಳೆಯಿಂದಬಂದ ಕಡ್ಡಿಗಳು<br>☐ 3. Dung cakes/ಬೆರಣಿ<br>☐ 4. Coal/ coke/ lignite/ ಕಲ್ಲಿದ್ದಲು, ಕೋಕ್, ಲಿಗ್ನೇಟ್<br>☐ 5. Charcoal/ ಕಲ್ಲಿದ್ದಲು<br>☐ 6. Kerosene/ಸೀಮೆಎಣ್ಣೆ<br>☐ 7. Electricity/ವಿದ್ಯುತ್<br>☐ 8. LPG/ ಗ್ಯಾಸ್<br>☐ 9. Bio-gas/ ಜೈವಿಕ ಇಂಧನ

G1. Do you wash your hands before preparing a meal?<br>ನೀವು ಅಡುಗೆ ಮಾಡುವ ಮುಂಚೆ ನಿಮ್ಮ ಕೈಗಳನ್ನು ತೊಳೆಯುತ್ತೀರಾ ☐ 1. Yes/ ಹೌದು ☐ 2. No/ ಇಲ
G2. Does your child wash hands before eating?<br>ನಿಮ್ಮ ಮಗು ಊಟ ತಿನ್ನುವ ಮೊದಲು ಕೈ ತೊಳೆಯುತ್ತದ? ☐ 1. Yes/ ಹೌದು ☐ 2. No/ ಇಲ

	Question	Choose the appropriate answer
F5	How much agriculture land does this household own?<br>*ನಿಮಗೆ ಕೃಷಿ ಜಮೀನು ಎಷ್ಟಿದೆ?*	☐ 1. 5acres or more/*೫ ಎಕರೆಗಿಂತ ಹೆಚ್ಚು*<br>☐ 2. 2 to 4.9 acres/ *೨ರಿಂದ ೫ ಎಕರೆ*<br>☐ 3. < 2 acres /unknown acreage<br>(*2 ಎಕರೆಗಿಂತಕಡಿಮೆ* / ಗೊತ್ತಿಲ್ಲ)<br>☐ 4. No land/ *ಜಮೀನು ಇಲ್ಲ* *(If 'No land' goto F7)*
F6	Out of this land, how much is irrigated?/ *ಇಷ್ಟು ಜಮೀನಿನಲ್ಲಿ ಎಷ್ಟು ನೀರಾವರಿ ಜಮೀನು ಇದೆ*	☐ 1. All / Some *ಎಲ್ಲಾ/ಸ್ವಲ್ಪ*<br>☐ 2. None/Don't know<br>*ಯಾವುದು ಇಲ್ಲ*/ ಗೊತ್ತಿಲ್ಲ
F7	Does this household own any livestock? ನಿಮ್ಮ ಮನೆಯಲ್ಲಿ ಯವುದಾದರು ಜಾನುವಾರುಗಳು ಇದೆಯೇ?	☐ 1. Yes/ ಹೌದು<br>☐ 2. No/ ಇಲ್ಲ

	Question	Choose the appropriate answer
F8	What is the main source of lighting for your household? ನಿಮ್ಮ ಮನೆಯಲ್ಲಿ ಬೆಳಕಿಗಾಗಿ ಯಾವುದನ್ನು ಉಪಯೋಗಿಸುತ್ತೀರಾ?	☐ 1. Electricity/ವಿದ್ಯುತ್<br>☐ 2. Kerosene /ಸೀಮೆಎಣ್ಣೆ<br>☐ 3. Gas/ಗ್ಯಾಸ್<br>☐ **4.** Candle/oil ಮುಂಬತ್ತಿ /ಎಣ್ಣೆ
F9	What is the main source of drinking water for members of your household?/ ನಿಮ್ಮ ಮನೆಯಲ್ಲಿರುವವರಿಗೆ ಕುಡಿಯುವ ನೀರಿಗಾಗಿ ಯಾವ ವ್ಯವಸ್ಥೆ ಇದೆ?	• Piped water (ನಲ್ಲಿಯ ಮುಖಾಂತರ)<br>☐ 1. Residence/ yard/ Plot (ಮನೆ, ಹೊರಗೆ, ಮನೆಯ ಅಗತ್ಯವಿರುವ ಕಡೆ)<br>☐ 2. Public tank (ಸಾರ್ವಜನಿಕ ನಲ್ಲಿ)<br>• Ground water/ ಅಂತರ್ಜಲ<br>☐ 3. Hand pump at residence/yard/plot (ಹ್ಯಾಂಡ್ ಪಂಪ್)<br>☐ 4. Public hand pump (ಸಾರ್ವಜನಿಕ ಹ್ಯಾಂಡ್ ಪಂಪ್)<br>• Well water (ಬಾವಿ ನೀರು)<br>☐ 5. Residence, yard, plot (ಮನೆಯ ಒಳಗೆ)<br>☐ 6. Covered well (ಮುಚ್ಚಿದ ಬಾವಿ)<br>☐ 7. Open well (ತೆರೆದ ಬಾವಿ)<br>☐ 8. Public well (ಸಾರ್ವಜನಿಕ ಬಾವಿ)<br>• Surface water<br>☐ 9. Spring (ಜಿಗಿ/ಚಿಮ್ಮು)<br>☐ 10. River/Stream (ಹರಿಯುವ ನೀರು)<br>☐ 11. Pond/Lake (ಕೆರೆ)<br>☐ 12. Dam (ಸಂಗ್ರಹವಾಗಿರುವ ಕೆರೆ)<br>• ☐ 13. Rain water (ಮಳೆಯ ನೀರು)<br>• ☐ 14. Tanker truck (ಹೊರಗಡೆಯಿಂದ ನೀರು ತರಿಸುವುದು)
F10	What kind of toilet facility does your household have?<br><br>ನಿಮ್ಮ ಮನೆಯಲ್ಲಿ ಯಾವ ರೀತಿಯ ಶೌಚಾಲಯ ವ್ಯವಸ್ಥೆ ಯಿದೆ?	• Flush toilet/ Latrine (ಫ್ಲಶ್/ನೀರಿನ ವ್ಯವಸ್ಥೆ ಇರುವ ಶೌಚಾಲಯ)<br>☐ 1. Own flush toilet (ಸ್ವಂತ)<br>☐ 2. Shared flush toilet (ಹಂಚಿಕೆಯ)<br>☐ 3. Public flush toilet (ಸಾರ್ವಜನಿಕ)<br>• Pit toilet/ Latrine (ಇಂಗು ಗುಂಡಿ)<br>☐ 4. Own pit toilet (ಸ್ವಂತ)<br>☐ 5. Shared toilet (ಹಂಚಿಕೆಯ)<br>☐ 6. Public toilet (ಸಾರ್ವಜನಿಕ)<br>• ☐ 7. No facility available *(Goto to G4)* (ಶೌಚಾಲಯ ಇಲ್ಲ)

PID | | | |

G3	Does this child use the toilet routinely?<br>ನಿಮ್ಮ ಮಕ್ಕಳು ನಿರಂತರವಾಗಿ ಶೌಚಾಲಯ ಬಳಕೆ ಮಾಡುತ್ತಾರೆಯೆ?<br>☐ 1.Yes ☐ 2. No<br>1. ಹೌದು 2. ಇಲ್ಲ	G4	Does your child wear footwear when going out? ನಿಮ್ಮ ಮಗು ಹೊರಗೆ ಹೋಗುವಾಗ ಚಪ್ಪಲಿಗಳನ್ನು ಧರಿಸುತ್ತದೇಯೇ?<br>☐ 1.Yes ☐ 2. No<br>1. ಹೌದು 2. ಇಲ್ಲ

F11	Does the household own any one of the following:<br>ನಿಮ್ಮ ಮನೆಯಲ್ಲಿ ಈ ಕೆಳಕಂಡ ವಸ್ತುಗಳು ಇವೆಯೇ?	Yes / No<br>ಹೌದು/ ಇಲ್ಲ<br>(Circle if owned)
1.	A Bed / ಹಾಸಿಗೆ	Yes / No
2.	A Pressure cooker / ಕುಕ್ಕರ್	Yes / No
3.	A Chair / ಕುರ್ಚಿ	Yes / No
4.	A Cot / ಮಂಚ	Yes / No
5.	A Table /ಬೆಂಚು	Yes / No
6.	A Clock / ಗೋಡೆ ಗಡಿಯಾರ	Yes / No
7.	A Wrist watch / ಕೈಗಡಿಯಾರ	Yes / No
8.	A Cycle / ಬೈಸಿಕಲ್	Yes / No
9.	A Radio/transistor / ರೇಡಿಯೋ	Yes / No
10.	A Tailoring machine / ಹೊಲಿಗೆ ಯಂತ್ರ	Yes / No
11.	A landline/mobile phone / ದೂರವಾಣಿ	Yes / No
12.	A Fridge / ರೆಫ್ರಿಜಿರೇಟರ್	Yes / No
13.	A Black & white TV / ಕಪ್ಪು ಬಿಳುಪು ಟಿ.ವಿ	Yes / No
14.	A Colour TV / ಬಣ್ಣದ ಟಿ.ವಿ	Yes / No
15.	A Two wheeler / ಸ್ಕೂಟರ್, ಮೋಟಾರ್	Yes / No
16.	A Car / ಕಾರ್	Yes / No
17.	A Water pump/ ನೀರಿನ ಬಾವಿ	Yes / No
18.	A Bullock cart / ಎತ್ತಿನ ಗಾಡಿ	Yes / No
19.	A Tractor / ಟ್ರ್ಯಾಕ್ಟರ್	Yes / No

H. Anthropometric details:

Child	Birth Weight (kg) :	
	Weight (kg):	
	Height/Length (cm):	
	Mid upper arm circumference (cm):	
	Head circumference (cm):	

I. Laboratory investigations: sample collection checklists

I1. Child:

Sl.no.	Sample	Sample collected		Sample volume
1.	Blood : 2 ml EDTA vacutainer (purple top)	☐ 1.Yes	☐ 2. No	ml
2.	Blood :1 ml Serum vacutainer (yellow top)	☐ 1.Yes	☐ 2. No	ml
3.	Urine:10 ml urine in sterile urine container	☐ 1.Yes	☐ 2. No	ml

I2. Mother:

Sl.no.	Sample	Sample collected		Sample volume
1.	Blood : 1 ml EDTA vacutainer (purple top)	☐ 1.Yes	☐ 2. No	ml
